# Prognostic Significance of Multifactorial Analysis in Complex Cervical Aero-Digestive Trauma Cases

**DOI:** 10.3390/medicina60020238

**Published:** 2024-01-30

**Authors:** Florentina Severin, Radu Danila, Andrei Nicolau, Anisia Iuliana Alexa, Raluca Olariu, Ștefan Roșca, Octavian Dragos Palade, Florin Mocanu, Mihail Dan Cobzeanu, Bogdan Mihail Cobzeanu

**Affiliations:** 1 Surgery Department, “Grigore T. Popa” University of Medicine and Pharmacy, 700115 Iasi, Romania; florentina-s-severin@umfiasi.ro (F.S.); radu.danila@umfiasi.ro (R.D.); nicolau.andrei@umfiasi.ro (A.N.); anisia-iuliana.alexa@umfiasi.ro (A.I.A.); raluca.olariu@umfiasi.ro (R.O.); mihail.cobzeanu@umfiasi.ro (M.D.C.); bogdan-mihail.cobzeanu@umfiasi.ro (B.M.C.); 2 Clinical Departement, “Dunarea de Jos” University, 800008 Galati, Romania; stefan.rosca@ugal.ro

**Keywords:** complex aerodigestive cervical trauma, predictable risk factors, multiple health risk factors, trauma prognosis, public health problem

## Abstract

*Background and Objectives*: In the context of complex aerodigestive cervical traumas, the prognosis and outcome heavily depend on risk factors, particularly injuries to the larynx, trachea, major digestive tissues, cervical vertebrae, and vascular structures. With the increasing prevalence of trauma as a public health concern, there is a pressing need for epidemiological research and the implementation of preventative measures. The purpose of this research is to establish the profile of the predictable impact factors that determine the prognosis of patients with complex cervical trauma. *Methods and Methods*: The study group consisted of 106 patients with complex cervical trauma pathology developed by various mechanisms such as car accidents, home-related accidents, aggression, gunshot wounds, and self-inflicted attempts, resulting in hospitalization in the E.N.T. Clinic at “St. Spiridon” Iași Hospital, from 2012 to 2016; medical records were the source of the collected data. *Results*: Hemodynamic instability upon admission associated with age, muscle and laryngeal injuries, and anemia were identified as negative prognostic factors. Additionally, the utilization of imaging-based paraclinical investigations for diagnosing traumatic lesions emerged as a positive prognostic factor in managing this pathology. The management of penetrating cervical trauma remains a subject of debate, with some advocating for surgical exploration beyond the platysma layer in all cases, while others argue for a more selective conservative approach due to a high rate of negative explorations. *Conclusions*: The statistical evaluation of epidemiological, clinical, lesion, paraclinical, and therapeutic parameters is needed to establish predictable risk factors in the prognosis of complex aerodigestive cervical trauma.

## 1. Introduction

Due to the complexity of the anatomical region of the neck, traumatic interests of all structures may occur, including aerodigestive, musculofascial, vascular, endocrine, or nervous. For this reason, the accurate diagnosis of complex cervical trauma is a challenge and can be extremely difficult. Therefore, we cannot forget that a rapid, unfavorable evolution toward death may accompany the diagnostic errors encountered in certain situations.

The standard of investigation procedures in cervical trauma cases depends on whether or not there are criteria for hemodynamic and neurological stability [1]. The unstable patient is provided with respiratory tract concomitant treatment with cervical surgery; the postoperative care of hemodynamic and neurological stability allows for supplementary endoscopic and imaging investigations. The symptomatic stable patient is subjected to endoscopic imaging and angiographic interventional investigations to establish the lesion distribution and the following development of healing conduct [2].

The overseeing of cervical trauma has been a topic of great interest and controversy. The critical surgical assessment of all cervical wounds originates from the two world wars. The development of the emergency medical system and paraclinical examinations has enhanced worldwide data on the mortality and morbidity of this pathology. Instead, the current otorhinolaryngological opinion has issued a hypothesis for the last five years, with already quite a few disciples, in support of selective surgical management. Diagnostic explorations are necessary, including panendoscopy, angiography, computed tomography, and esophagography. Explorations are recommended only in respiratory and hemodynamically stable patients. This approach to management is preferable to be performed only in hospital units with the necessary logistical support [3,4,5,6]. The mortality and morbidity of negative surgical examinations are rarely higher than the traumatic injuries themselves [5,6,7,8,9,10,11,12,13,14,15]. In our research, we collected information from medical records regarding epidemiological elements, clinical and paraclinical exploration, as well as an indication for surgical exploration, processed by statistical tools to establish the sensibility and sensitivity of each parameter to establish the type of risk factor, either positive or negative, involved in determining the prognosis of complex aerodigestive cervical trauma.

The manuscript is structured as follows: Section 2 presents the materials and methods utilized for conducting this study. The acquired results are organized in Section 3. This is followed by Section 4, where we analyze our findings and their interpretations of the existing literature and draw a conclusion in Section 5.

## 2. Materials and Methods

The group consisted of 106 patients with complex cervical trauma pathology resulting from multiple mechanisms such as car accidents, home-related accidents, aggression, gunshot wound trauma, and self-inflicted attempts, admitted to the E.N.T. Clinic at “St. Spiridon” Hospital of Iași from 2012 to 2016; medical records were the source of the collected data. The patient information gathered for this research was maintained confidentially. This research study underwent analysis and subsequently received approval by the Ethics Committees of both “Grigore T. Popa” University of Medicine and Pharmacy and the “St. Spiridon” Emergency Hospital of Iasi. This study was partially limited because information on particular patients was incomplete due to the retrospective aspect and the lack of consistency in describing the investigations performed on patients, which could harm the data identified in this case. The data were collected from the IBM SPSS Statistics 26.0 database (IBM corp, Armonk, NY, USA). The overall exclusion criteria were aged under 18, with the patient’s decision not to take part or the presence of a history of cervical trauma, but without pathological injuries or only superficial injuries without association with symptoms.

## 3. Results

Our research included 106 patients, and the three dominant identified mechanisms were self-inflicted, aggression, and accidental. All the information we collected for this study was studied statistically using the logistic regression method, which we used to fit a regression model when the response variable was binary, and we created an ROC curve of the SPSS data to study our parameters. To obtain the score, we measured the area under the ROC curve. The ROC AUC score shows how well the classifier distinguishes positive and negative classes. It can take values from 0 to 1. A higher ROC AUC indicates better performance. A good prognosis is defined as healing all lesions in the absence of inflammatory or functional aerodigestive complications (restitutio ad integrum). A poor prognosis means the need for surgical intervention, the presence of functional aerodigestive complications, or even death.

**Figure 1:**
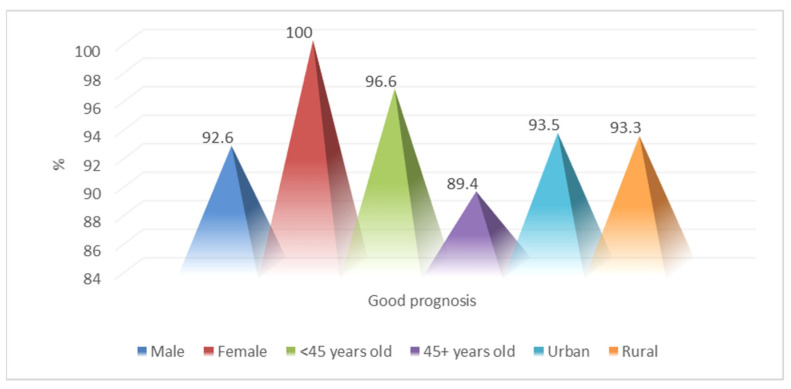
The epidemiological characteristics of patients with a favorable prognosis.

Based on these factors, favorable evolution was noticed in 99 of the patients (93.4%), more frequently in females (92.6% vs. 100%; *p* = 0.207), the age group of under 45 years (96.6% vs. 89.4%; *p* = 0.134) and the results were approximately equal by origin (93.5% vs. 93.3%; *p* = 0.976) (Figure 1).

**Figure 2:**
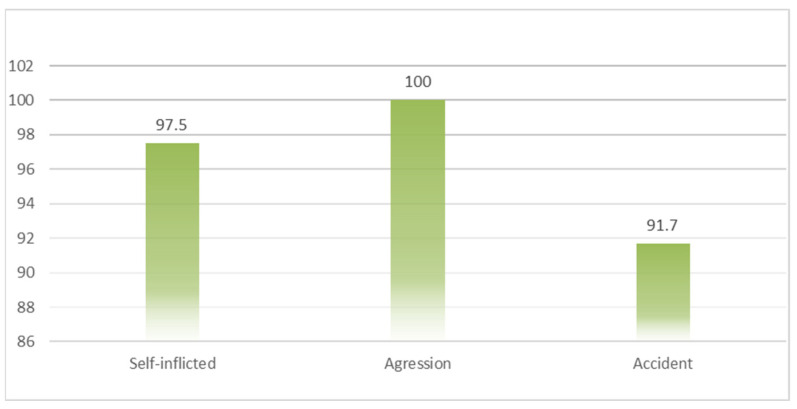
Distribution of cases with favorable prognosis according to the lesion mechanism.

A good prognosis was recorded by all patients with aggression (100%) and 97.5% of those with self-inflicted lesion mechanisms, whereas, of the selected patients with an accidental lesion mechanism, only 91.7% had a favorable prognosis (*p* = 0.022) (Figure 2).

Seven patients from the selected case series had an unfavorable prognosis, of which five died. The epidemiological characteristics identified in patients with an unfavorable prognosis were all male, with three patients over 45 years old, three patients from rural areas, and four patients with cervical trauma induced by the self-inflicted lesion mechanism.

Multiple analyses showed that age could be a significant predictor risk factor (*p* = 0.048) for an unfavorable prognosis (Table 1).

**Figure 3:**
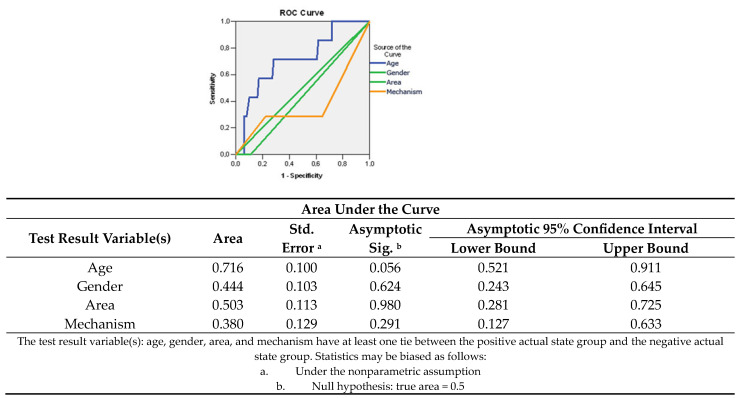
Sensitivity/specificity balance of epidemiological data for determining the negative prognostic.

This aspect was also confirmed by plotting the R. The O.C. curve showed that age is a good predictor factor of unfavorable prognosis with a probability of 71.6% (A.U.C. = 0.716; 95% CI: 0.521–0.911) (Figure 3).

**Figure 4:**
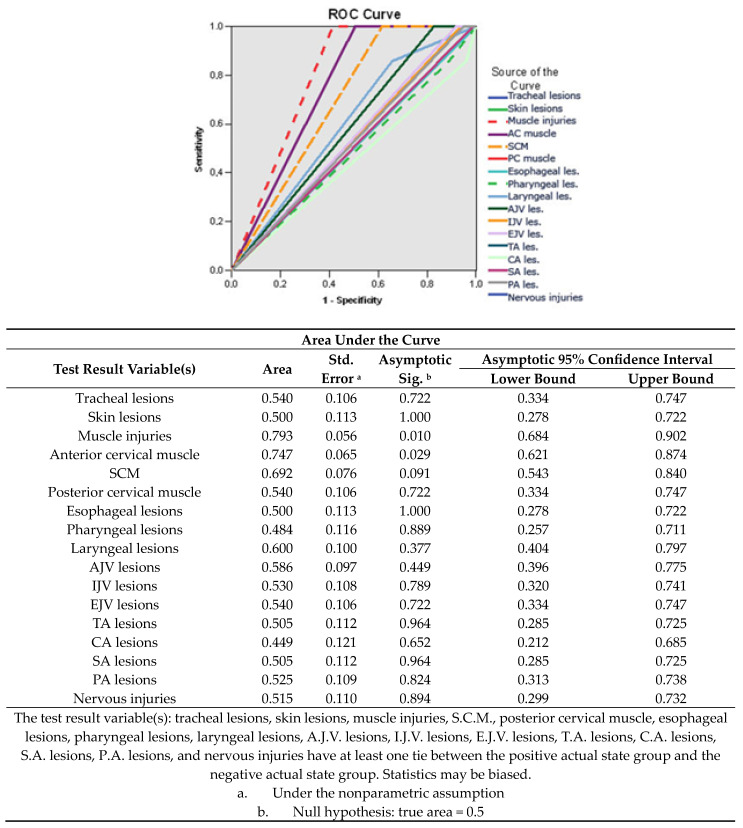
The sensitivity/specificity balance of the types of lesions for determining the unfavorable prognosis.

Plotting the ROC curve in terms of the effect of the visceral injuries caused by traumatic action on the prognosis factors identifies the total muscle injuries (AUC = 0.793; 95% CI: 0.334–0.747) of the anterior cervical muscles (AUC = 0.747; 95% CI: 0.621–0.874), the lesions of the sternocleidomastoid muscle (AUC = 0.692; 95% CI: 0.543–0.840) and laryngeal lesions (AUC = 0.600; 95% CI: 0.278–0.722), as a risk factor in determining the unfavorable prognosis (Figure 4).

**Figure 5:**
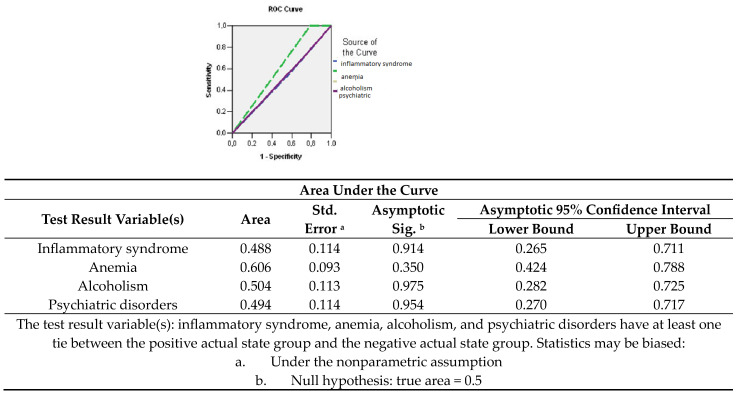
Sensitivity/specificity balance of comorbidities and blood parameters for determining an adverse prognosis.

By tracing the R.O.C. curve and depending on the influence of changes in blood parameters and associated pathologies, it was highlighted that the presence of anemia influenced the unfavorable prognosis as a negative risk factor (A.U.C. = 0.606; 95% CI: 0.424–0.788) (Figure 5).

**Figure 6:**
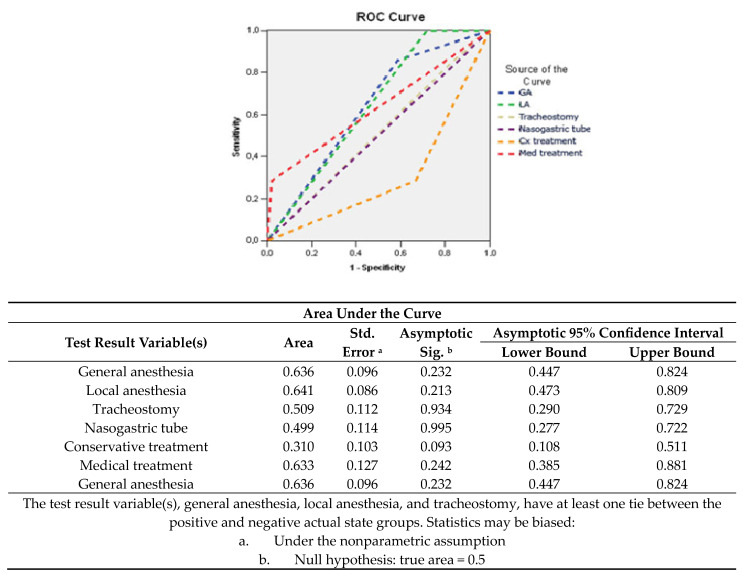
Sensitivity/specificity balance of comorbidities and blood parameters for determining an unfavorable prognosis.

Determining an unfavorable prognosis may include risk factors such as the need for surgical exploration under general anesthesia (AUC = 0.636; 95% CI: 0.447–0.824) or local anesthesia (AUC = 0.641; 95% CI: 0.473–0.809) and drug treatment (AUC = 0.633; 95% CI: 0.385–0.881) (Figure 6).

**Figure 7:**
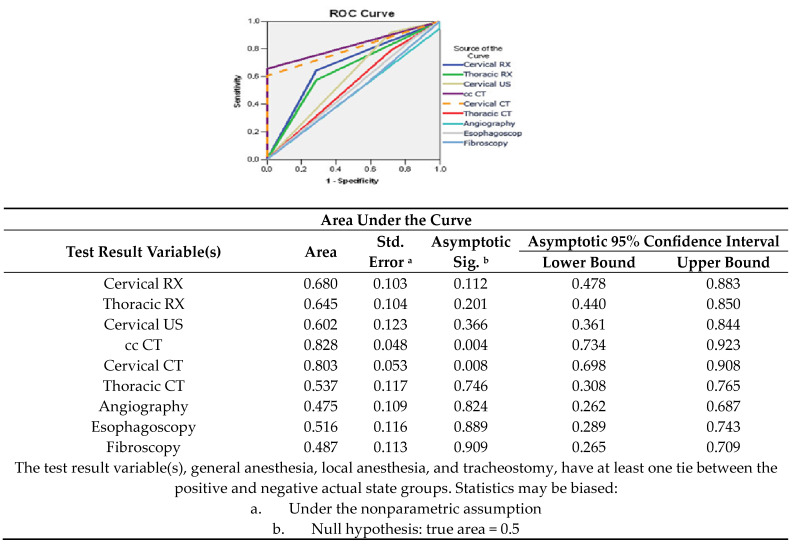
Balance sensitivity/specificity of paraclinical diagnosis methods for determining a favorable prognostic.

In the analyzed case studies, the following investigations were good predictors of risk factors for a favorable prognosis (Figure 7):

- Craniocerebral CT, with a sensitivity of 65% and a specificity of 98% (AUC = 0.828; 95% CI: 0.744–0.923);
- Cervical CT, with a sensitivity of 60% and a specificity of 98% (AUC = 0.803; 95% CI: 0.698–0.908);
- Cervical radiography, with a sensitivity of 62% and a specificity of 75% (AUC = 0.680; 95% CI: 0.478–0.883);
- Chest radiograph, with a sensitivity of 58% and a specificity of 75% (AUC = 0.645; 95% CI: 0.440–0.850);
- Cervical ultrasound, with a sensitivity of 70% and a specificity of 55% (AUC = 0.602; 95% CI: 0.361–0.844).

**Figure 8:**
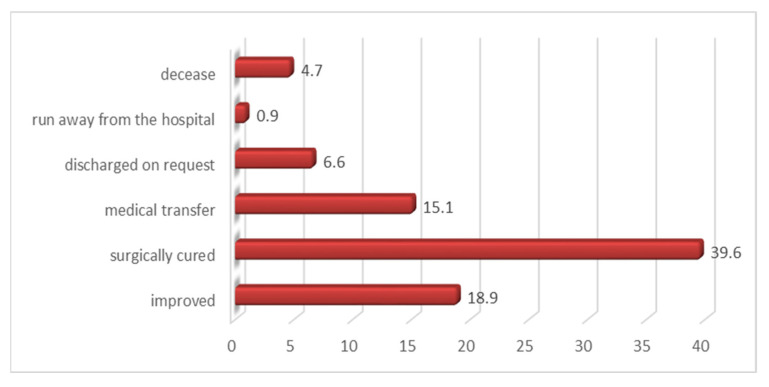
Distribution of cases with good prognosis according to the lesion mechanism.

A share of 39.6% of patients cured postoperatively and 18.9% with an improved status at discharge was noted in our study (Figure 8).

## 4. Discussion

Based on the information collected from medical records regarding epidemiological elements, clinical and paraclinical exploration, as well as the indication for surgical exploration, the achieved results established the sensibility and sensitivity of each parameter to specify the type of risk factor, either positive or negative, involved in determining the prognosis of complex aerodigestive cervical trauma. We interpreted them according to our findings and existing studies in the literature.

The medical management of complex cervical trauma has turned into a conservative approach over time. The necessity for surgical examinations in patients with penetrating traumatic lesions of the aerodigestive tract or large vessels should be founded on clinical evidence, which is a credible indicator for an open examination [15].

The count of medical and medical–surgical specialties engaged and the diagnostic approach of patients with traumatic cervical injuries require the development and implementation of a multidisciplinary procedure with national applicability. In addition, it could support the indications of paraclinical diagnostic procedures, such as flexible nasopharyngolaryngeal fibroscopy, plain radiography, esophagography, computed tomography, and clear indications of surgical versus conservative treatment.

Evidence of the presence of prevertebral air on cervical or thoracic radiography, profile incidence, or the extravasation of a contrast agent may confirm a suspicion of digestive perforation with sensitivity ranging from 48 to 100%, with inferior results for the hypopharynx [16,17,18]. In our study, the use of cervical or chest radiographs had a sensitivity and specificity in the range of 58–75%, with data being consistent with existing studies.

Doppler imaging was supported as a non-invasive option to conventional arteriography, with a sensitivity of 91% and a specificity of 99% to detect vascular lesions in a study consisting of a succession of 82 cases [19]. The limitations of this method include the fact that the performance and proper interpretation of an ultrasonographic examination are operator-dependent; it is unlikely to provide helpful information on lesions of non-vascular structures or the path of a stabbing instrument. This low occurrence can lead to a decrease in the suspicion rate and the subsequent postponement of diagnosis. Prolonging the diagnosis from 12 to 24 h can increase the extravasation of bacteria, saliva, and gastric reflux, leading to lesion extension. When an experienced technician performs this scan, the sensitivity of the ultrasound compared to a conventional angiographic scan as a reference technique is 90% to 95% for lesions that require interventional treatment. This examination may omit lesions with preserved flow, such as intimate denudation or pseudoaneurysms [19,20,21,22,23]. In our study, the Doppler ultrasound had a sensitivity of 70% and a specificity of 55% and was shown in 10 patients out of 106 with injuries caused by accidental mechanisms.

Mazolewski et al. evaluated the results of tomographic investigations compared to the elements identified intraoperatively. They concluded that computed tomography has a high sensitivity and specificity of approximately 100% and 91%, respectively, for identifying the significant lesions of zone II traumas [24]. Another prospective study looked at computerized angiography as an initial imaging method for assessing stable patients with penetrating neck trauma. Inaba et al. revealed a sensitivity of 100% and a specificity of 93.5% in identifying aerodigestive and vascular lesions. Due to these characteristics, the imaging method decreased the number of routine cervical surgical examinations performed on stable patients who did not require emergency surgical treatment [25,26,27]. 

In 2006, Osborn et al. published a retrospective study conducted in the Trauma Center of Portland, Oregon, U.S.A., between 2000 and 2005. This study highlights the role of computed tomography and angiography in managing patients with penetrating cervical trauma in terms of indicating surgical treatment. Computer angiography proves its specificity in diagnosing vascular lesions while diagnosing aerodigestive lesions has proven challenging. Cervical emphysema and crackling suggest upper aerodigestive tract lesions and require further investigation. C.T. scans with or without direct laryngoscopy or bronchoscopy are sufficient to diagnose laryngeal or laryngeal–tracheal lesions. Classically, digestive lesions are diagnosed via esophagography, a 70–80% specific method, while a C.T. and angiographic examination revealed a sensitivity and specificity of diagnosis of about 100%. Although traumatic cervical and gastrointestinal lesions are rare, failing to diagnose them is accompanied by significant mortality and morbidity. The indication for the surgical exploration of penetrating cervical trauma after completing a preoperative C.T. is an approach that is somewhat related to the affected cervical area and the lesion described to avoid formal and unnecessary surgical explorations [25]. Considering the studies presented above, compared to the case of our research, the use of computed tomography exploration registered a sensitivity and specificity of 60% and 98%, respectively, falling within the value range of the exposed studies. Advantages include rapid availability and minimal invasion compared to conventional arteriography. A CTA can also be executed quickly, with most scans lasting between 2 and 3 min. Unlike a cervical Doppler ultrasound, C.T.A. is not operator-dependent and produces high-quality images that can be explained by treating clinical staff and radiologists. Furthermore, C.T.A. can demonstrate damage to structures separate from the arterial shaft, such as the aerodigestive tract. The trajectory resulting from a penetrating cervical trauma caused by a stabbing device can also often be viewed on a C.T.A. scan to further elucidate the structures that may be exposed to the risk of injury, and, therefore, further investigation is required [18]. 

Srinivasan et al. identified flexible esophagoscopy as 100% sensitive and 92.4% specific [28,29]. In the case of the suspicion of penetrating lesions, rigid and flexible esophagoscopy was used in 11 patients in 11.3% of all cases, while esophagography was not used as a diagnostic method.

In their 2016 study, Kasbekar et al. identified the use of nasopharyngeal–laryngeal fibroscopy in the lesion diagnosis of 50% of three patients with cervical trauma. The data in our study had a 45.3% close value compared to existing studies [30].

In our study, the reassessment of patients after discharge was 14.1% as opposed to other similar studies, in which the range was between 30 and 40%. Incomplete patient data from studies related to patient re-evaluation have been sources of influence on final data processing, which is one of our research limitations [31,32,33].

## 5. Conclusions

In our study, the indication for paraclinical imaging was influenced by the need to secure the airway via orotracheal intubation or tracheostomy and hemodynamic status. The high sensitivity and specificity of imaging techniques, such as computed tomography, arteriography, simple radiographs, and cervical ultrasound, are indicated in the specific lesion diagnosis in complex cervical traumatology. The use of imaging paraclinical explorations diagnosing traumatic lesions is a positive risk factor for the prognosis of this pathology. The negative risk factors for the prognosis identified in the complex aerodigestive traumatic pathology of the neck are age (71.65%), muscle and laryngeal injuries, anemia, and the need for surgical exploration. Identifying the risk factors involved in the prognosis of complex cervical aerodigestive trauma could be helpful in surgical or conservator therapeutic management or in the future to prevent a real public health problem due to the ongoing growth of the incidence, applying cost-effective paraclinical investigations, all in the patient’s best interest.

## Figures and Tables

**Table 1:** Multiple linear regression—prognosis-dependent variables.

Model	R	R Square	Adjusted R Square	Std. Error of the Estimate	Change Statistics				
					R Square Change	F Change	df1	df2	Sig. F Change
1	0.193 (a)	0.037	0.028	0.246	0.037	40.008	1	104	0.048
2	0.211 (b)	0.044	0.026	0.246	0.007	0.789	1	103	0.377
3	0.212 (c)	0.045	0.017	0.247	0.001	0.076	1	102	0.784
4	0.232 (d)	0.054	0.017	0.247	0.009	0.950	1	101	0.332
5	0.234 (e)	0.055	0.007	0.249	0.001	0.059	1	100	0.809

a Predictors: (constant), age; b predictors: (constant), age, gender; c predictors: (constant), age, gender, area; d predictors: (constant), age, gender, area, mechanism; e predictors: (constant), age, gender, area, mechanism, topography; and f dependent variable: prognosis.

## Data Availability

The data presented in this study are available on request from the corresponding author.
